# From trainee to tenure-track: ten tips

**DOI:** 10.1186/s13059-015-0691-4

**Published:** 2015-06-24

**Authors:** Tuuli Lappalainen

**Affiliations:** New York Genome Center, 101 Avenue of the Americas, New York, NY 10013 USA; Department of Systems Biology, Columbia University, New York, NY 10032 USA

During the past year, after starting as a tenure-track faculty member, a number of younger scientists have asked me: “How did you do get there?” The internet is full of horror tales of academic careers, but the story I want to tell you is the opposite — how the years as a trainee in the world of academia can lead to a great job. My intention is not to brag, because I know that I have been very lucky. Indeed, I am still a bit astonished that a small-town girl from eastern Finland ended up all the way here. In this article, I want to contribute to the discussion of career development in the academic environment by reflecting on lessons that I have learned about succeeding as a young academic researcher.

There is no straightforward recipe for success, but neither is it a random process, nor are those of us who make it to a faculty position necessarily blessed with off-the-charts intellectual abilities. I see success as a mixture of hard work, support, luck, strategy, persistence, talent and personality. You may need to do some extra work if you do not come from the inner circle of top universities — I did not — but those barriers are there to be broken. If you doubt whether an academic career is worth the effort, the world is full of alternative careers that can be just as exciting, better paid or less demanding. People who choose to leave after a PhD or after a postdoc are not failures. But especially in human genomics, the job market is not totally impossible, and there is opportunity in the field of academia to do groundbreaking science.

So, what would be my advice for graduate students and new postdocs in genomics who want to aim high in their academic career? In the following, I present my top ten tips:

1. *Work hard*
  If you know a scientist who has made it to the very top with a nine-to-five, Monday-to-Friday mentality, please let me know because I would be curious to meet such a person one day. The last time I got home from work and casually thought “five hours until bedtime, I wonder what’s on TV” was in 2007! And I’m not an exception. You know those emails that your boss sends you at 3:00 a.m.? It is not a coincidence that he is the boss and that he is up working at the wee hours. Of course, it is not all about the hours: being able to work efficiently is an essential skill. Many of the most successful of us work both very efficiently and very long hours.
2. *Have fun*
  If you are not genuinely interested in what you do and able to appreciate the people around you, it will show. Most people are not good at faking it — and I am so bad that I do not even try. If my talks and papers are exciting, it is because I am excited myself. I think that my consortium projects turned out well because I *honestly* found the interactions fruitful instead of an unproductive political game. Meeting senior scientists is a fantastic opportunity to learn from them and have an interesting discussion, and not a situation where you should pull out a mask to make a good impression. Not everything is fun, but if you feel all the time that you need to pretend, maybe you should find another project or even another job. An academic salary is not high enough compensation for work that you don’t enjoy. You are unlikely to get very far if your heart is not in it.
3. *Do good work and get it done*
  People remember extremely well who delivers and who does not. The most common mistake, especially for young scientists, is having very big, perfectionistic plans, but in the end *nothing* gets done in a reasonable time-frame. Simple solutions can be great. At the other end of the spectrum are those who like to talk but cannot be bothered to do the actual work. Whatever you do, invest in impeccable presentation of the work in papers and talks. Science needs to be rigorous and ambitious, but better *done* than *perfect*.
4. *Network, network, network*
  Personal relationships matter in every human endeavor. No matter how great your papers are, you will have a hard time building a great career if no one has ever met you. This might not be fair or the way science should be, but that is the way it is. Work on collaborative projects, go to conferences and leverage the networks of your principal investigator (PI), coworkers and friends. Be on Twitter. Drink beer with colleagues. Talk to people both above and below your academic rank. Those who are a couple of years ahead of you are an irreplaceable source of practical advice and support. Be nice and play fair — the word will spread if you screw over collaborators or act like a jerk.
5. *Choose your area of expertise*
  Try to figure out what is the next big thing within your broader field, and get into a pioneering lab that is doing it right now. Functional population genomics and population-scale RNA-sequencing were in their infancy when I started my postdoc, but by the time I was applying for faculty jobs, there was a lot of demand for this expertise. That was my strategy: in addition to having a genuine interest, I guessed that these areas would be big. If you have a burning passion for something untrendy, it might be worth the risk, but make it a conscious risk.
6. *Join a great lab*
  My postdoc advisors, Manolis Dermizakis in Geneva and Carlos Bustamante at Stanford, gave me not only scientific guidance but also the opportunity to work on great projects, encouragement and recognition. I worked *with* them, not *for* them. You want an advisor who invests in their trainees’ success rather than one who just uses them as a workforce. And how do you know that when you are applying for a job? Talk to people who know the PI, including their current and past trainees, and listen to the grapevine and your gut feeling. Both established and new labs can be good choices. Few PhDs have the track record to walk into the best labs just like that — neither did I — but getting your own fellowship will open many doors.
7. *Build a diverse skill set*
  If you want to run a lab one day, you will need skills beyond those of fundamental scientific knowledge: project and time management, communication and public relations, grant writing, leadership and the networking skills already mentioned. Use your time as a trainee to get experience in these areas as well. Ask your PI to get you involved in grant writing, and find out what your experiments, data and computational resources are worth in real money. Learn the logistics of research projects. If you are interested in public outreach or scientific journalism, do it. But do not get lost in management tasks or the blogosphere — peer-reviewed papers still count the most.
8. *Have a life*
  You should work hard, but sacrificing your physical or mental health, all other areas of interest and personal relationships is simply not worth it, and no one will admire you for it. You can’t force yourself to be creative and efficient if you do not give your brain any time off, and you don’t want to end up celebrating your great achievements all by yourself because you have neglected your friends and family for too long. In the end, it is *your* life, and you will regret even the most fantastic career if you sacrifice other things you really want. I have made peace with myself by drawing the line regarding how much I will sacrifice, and accepting that this will take me as far as I should get.
9. *Challenge yourself*
  My philosophy is always to have one foot outside of my comfort zone, because that is how I learn and develop. When I get really comfortable and I feel like I really know what I am doing, it is time for the next challenge. Yes, it is scary. No, it is not easy. Yes, you will screw up sometimes. So what? Seize and create new opportunities and push new frontiers for yourself. Tackle the biggest problems you think you can solve. Take big problems as opportunities and the search for solutions as adventures.
10. *Be brave, persistent and patient*
  You probably feel small and stupid sometimes, at least if you are challenging yourself enough. But so do we all. I feel inadequate on a regular basis. It is good to be humble, but do not let that stop you from pursuing ambitious projects, having big career plans or opening your mouth when you have something to say. Lean in, even if you need to make it a conscious effort. If you are doing things right, you are taking risks and investing a lot in your career. That is going to make your road bumpy, and the rejections, failures and criticism will hurt. But there is *always* a way out somewhere, even when you can’t see it on the spot. As long as you have followed ethical guidelines, your career is never going to be over just because your paper was rejected, your code had a bad bug, you broke an instrument, you said something very stupid in an important professional situation, or someone dislikes you. I have experienced all of these — we all have. Finally, rushing through your career path might get you a job, but not necessarily The Job. Enjoy your time as a postdoc when you can focus on the science, and take the time that you need to have the papers and expertise that will put you in a great position on the job market.

These principles have worked well for me thus far, but there are probably many other viewpoints on how to not only survive in the academic sphere but how to thrive, achieve your personal goals, do great science and have fun at the same time. Getting a faculty job is just the beginning, and for me the biggest challenges are ahead. If I manage to do well on the tenure track, I promise to write about my experiences again six years from now.

